# Enhancing the Efficacy of Breast Cancer Immunotherapy Using a Smac-Armed Oncolytic Virus

**DOI:** 10.3390/cancers16193248

**Published:** 2024-09-24

**Authors:** Sijia Tang, Kristin V. Lyles, Yuzhen Wang, Daping Fan, Ming Luo

**Affiliations:** 1 Institute of Biomedical Sciences, Georgia State University, Atlanta, GA 30302, USA; stang13@student.gsu.edu; 2 Department of Chemistry, Georgia State University, Atlanta, GA 30302, USA; kvanmouwerik1@gsu.edu; 3 Department of Cell Biology and Anatomy, University of South Carolina School of Medicine, Columbia, SC 20209, USA; wang379@mailbox.sc.edu (Y.W.); daping.fan@uscmed.sc.edu (D.F.); 4 Center for Diagnostics and Therapeutics, Georgia State University, Atlanta, GA 30302, USA

**Keywords:** armed oncolytic virus, immunotherapy, triple negative breast cancer, Smac, pulmonary delivery, combination therapy, metastasis, tumor microenvironment

## Abstract

**Simple Summary:**

Immune checkpoint inhibitors (ICIs) are the most recent breakthrough in cancer treatments. Several drugs have been approved by the FDA including anti-PD-1 antibodies (Nivolumab, Pembrolizumab, and Cemiplimab). Their treatment is most effective for melanoma, non-small cell lung cancer, or bladder cancer. However, ICI response rates are very low for breast cancer patients, even for those with triple negative breast cancer (TNBC) who are expected to be more responsive to ICI treatment. Improved treatments are in demand for patient benefits. Our armed oncolytic virus is one therapeutic agent that can improve the outcome of TNBC treatment, especially in combination with ICIs, as demonstrated by this study.

**Abstract:**

It has been shown that the response rate of TNBC is dependent on the level of PD-L1 and the tumor microenvironment (TME). Approaches that alter the TME can improve the efficacy of ICIs. **Background**: We have engineered a Smac-armed oncolytic virus by inserting a Smac transgene into the genome of a vesicular stomatitis virus to generate VSV-S. Our previous study shows that the anticancer efficacy of VSV-S is more potent than that of wild-typed VSV in a subcutaneous TNBC mouse model. VSV-S treatment reverts the immunosuppressive TME by reducing MDSCs and TAMs, while increasing infiltration of neutrophils and CD8+ T cells. **Methods**: VSV-S was used to treat TNBC in an orthotopic mouse model, and in a combination therapy with an anti-PD-1 antibody to treat metastatic TNBC in a mouse model. Changes in the TME were evaluated. **Results**: In this current study, we show that neoadjuvant VSV-S treatment of primary orthotopic TNBC tumors in mice drastically lowered lung metastasis after surgical removal of the primary tumor, and significantly increased the survival rate. The mechanism of action and changes to the TME were delineated, among which one significant marker is the elevation of PD-L1 expression in tumors. In the TNBC lung metastasis mouse model, pulmonary treatment with VSV-S greatly enhanced the efficacy of ICI treatment. **Conclusions**: Our results suggest that the combination of oncolytic virus and ICI therapies has the potential to substantially improve the outcome of TNBC treatment.

## 1. Introduction

The WHO reports that 2.3 million women were diagnosed with breast cancer in 2022, and 685,000 deaths occurred globally. Breast cancer is the most prevalent cancer in the world today. With advances in treatment options, 5-year relative survival rates for breast cancer continue to improve, reaching more than 90% when all cases are combined [1]. However, the rate drops to about 30% for metastasized cases, especially for triple-negative breast cancer (TNBC). A reduction in metastasis and improvement of treatment for TNBC are urgent unmet medical needs.

One of the recent advances in treatment for TNBC is immunotherapy by immune checkpoint blockade [2]. Immune checkpoint inhibitors (ICIs), such as atezolizumab, a programmed cell death-ligand 1 (PD-L1) antibody, and pembrolizumab, a programmed cell death-1 (PD-1) antibody, have shown modest responses in clinical trials [3,4]. The only FDA-approved treatment is pembrolizumab in combination with neoadjuvant chemotherapy for high-risk, early-stage, TNBC [5], or in combination with chemotherapy for PD-L1-positive, locally recurrent inoperable or metastatic TNBC [6]. It is still a challenge to advance ICIs as a broadly efficacious treatment for TNBC.

TNBC irresponsive to ICI treatment may be derived from a number of different mechanisms [7]. One of the indicators is the level of PD-L1 expression in TNBC. Generally, TNBC has a relatively higher level of PD-L1 expression than other breast cancer subtypes [8]. The level of PD-L1 in TNBC cells has been shown to be positively correlated with the response to atezolizumab [9]. Manipulation of PD-L1 expression has been shown to alter responses to ICIs. In mice engrafted with human immune cells, the growth of MDA-MB-231 TNBC xenografts was reduced by treatment with an anti-PD-L1 antibody, given sequentially following cyclin-dependent kinase inhibitor SNS-032 dosing [10]. Cell surface PD-L1 expression was elevated by suboptimal SNS-032 dosing. Inhibition of mitogen-associated kinase TTK induced DNA damage and an increase in infiltration of CD8+ T cells, and, at the same time, upregulated the expression of PD-L1 in mouse TNBC cell line 4T) [11]. Combination treatment of a TKK inhibitor, BAY-1217389, and anti-PD-1 considerably increased inhibition of tumor growth in a subcutaneous 4T1 mouse model (*p* < 0.0001).

The presentation of immunogens and immunosuppressive tumor microenvironment (TME) in TNBC are also determining factors of ICI responses [12]. The correlation of neoantigen presence in tumors with infiltration of lymphocytes indicated that effective elimination of heterogenous neoantigen bearing tumor cells resulted from the high infiltration of lymphocytes in TNBC [13,14]. Tumor-associated macrophages (TAMs), such as a monocyte-derived lipid-associated macrophages, mediate immune suppression of immune checkpoint blockade response by inhibition of T-cell activation and proliferation [15]. The increase in myeloid-derived suppressor cells (MDSCs) in tumors also contributes to the creation of an immunosuppressive TME [16]. The induction or accumulation of granulocytic myeloid-derived suppressor cells (gMDSCs) enhanced suppression of antitumor immunity [17,18]. In the 4T1 tumor model, hyperoxia treatments relieved hypoxia levels in the lung TME and decreased the proportion of MDSCs in both the primary tumor and in the metastatic lung [19]. These findings suggest that reduction in immunosuppressive cells, including TAMs and MDSCs, in the TME can revert the suppression of immune checkpoint blockade in TNBC.

In our previous studies, we demonstrated that by insertion of the Smac expression cassette in the genome of vesicular stomatitis virus (VSV-S), the intracellular levels of Smac were restored in contrast with the wild-type VSV that eliminates the endogenous Smac during infection [20]. Apoptosis was significantly enhanced by VSV-S treatment of 4T1 cells and tumors in vivo. In addition, the infection of VSV-S also resulted in the elimination of MSDCs and TAMs in tumors, especially a reversed ratio of M2 to M1 types [21]. In this report, we explored the outcomes of VSV-S treatment in the orthotopic 4T1 model with regard to lung metastasis and pulmonary delivery of VSV-S for treatment of metastatic tumors.

## 2. Materials and Methods

### 2.1. Virus and Cells

VSV-S was constructed previously [20] and was propagated in HeLa cells and 4T1 cells. HeLa cells (ATCC) and EO077 and 4T1 cells (a gift from Dr. Wan) were grown in DMEM with 10% FBS. For virus infection, the cells were maintained in DMEM with 1% FBS. Cell viability was measured with a CCK-8 kit 24 h after infection. Plaque assays were carried out to determine the plaque forming unit (PFU) in HeLa cells. TCID50 assays were carried out in 4T1 cells and infection was evaluated by cytopathic effect. For animal studies, adapted VSV-S was propagated in 4T1 cells and concentrated by pelleting. The virus pellet was resuspended in PBS for use in animal studies.

### 2.2. Animal Studies

For orthotopic inoculation of breast cancer cells, on Day 0, 2 × 10^5^ 4T1 cells in 20 μL PBS were injected into each of the 4th pair of mammary fat pads of 8-week-old female BALB/c mice. Eighteen mice were randomly assigned to two groups, a vehicle (PBS) control group or a VSV-S group, with 9 mice in each group. On Day 14 and Day 16, 20 μL PBS or 2 × 10^6^ PFU VSV-S in 20 μL PBS was injected intratumorally (i.t.) into each tumor; and then, on Day 18, all tumors were completely surgically removed, along with tumor draining lymph nodes. The tumors and lymph nodes were freshly processed for flow cytometry, quantitative real-time PCR and Western blot analyses. The mice were monitored for survival until Day 34. When the mice died before or were euthanized at the endpoint (Day 34), the lungs were collected. The lungs were photographed and then fixed for further histological analysis.

To establish a metastasized model, Balb/c mice (6 weeks, female) were purchased from Jackson Laboratory (Bar Harbor, ME, USA) and acclimated for one week. On Day 0, the mice were injected intravenously (IV) in the lateral tail vein with 1.0 × 10^6^ of the 4T1 cells that were quantified with a Hausser Brightline hemacytometer (Horsham, PA, USA). The mice were then divided into 4 treatment groups (n = 5). On Days 5 and 7, the mice received 30 μL of either 3 × 10^6^ PFU VSV-S (VSV-S or group 1) and VSV-S + anti-PD1 (group 2) or PBS (anti-PD1 or group 3) and PBS (group 4) intranasally (IN). On Day 15 and every 4 days after that, the mice received an intraperitoneal (IP) injection of 60 μL per mouse of either 200 μg of anti-PD-1 antibody (antibody purchased from BioXCell, RMP1-14, Lebanon, NH, USA) for groups 2 and 3 or PBS for groups 1 and 4. The mice were monitored daily and were weighed every other day. The mice were euthanized if they lost significant body weight, had tumors greater than 2 cm in any one dimension, or turned necrotic.

The lungs were removed, perforated with PBS, then soaked in 10% neutral buffered formalin for at least 24 h before being embedded in paraffin.

The animal care procedures and experimental methods were approved by the Institutional Animal Care and Use Committee (IACUC) of the University of South Carolina or Georgia State University.

### 2.3. Flow Cytometry

Cell populations in the tumors and lymph nodes were analyzed using flow cytometry as previously described [22,23]. Briefly, cells were stained with anti-CD11c-APC (Biolegend 117310), anti-CD3-APC-Cy7 (Biolegend 100330), anti-CD8-Percp-Cy5.5 (Biolegend 100733), anti-I-A/I-E-FITC (Biolegend 107605), or anti-CD27-FITC (Biolegend 124207) in PBS containing 2% FBS for 30 min at 4 °C. The samples were washed twice with staining buffer and analyzed with flow cytometry using a BD FACS Aria II flow cytometer and CXP software version 2.2. Data were collected for 20,000 live events per sample.

### 2.4. Quantitative Real-Time PCR

Total RNA was isolated from tumor tissues and purified using Qiagen RNeasy Kits (Qiagen). Two micrograms of total RNA (2 μg) were then reverse transcribed to cDNA using iScript cDNA Synthesis Kit (Bio-Rad). Quantitative real-time PCR was conducted using iQ SYBR Green Supermix (Bio-Rad) on a CFX96 system (Bio-Rad) following the manufacturer’s instructions. All primers were synthesized by Integrated DNA Technologies. The relative amount of target mRNA was quantified using the comparative threshold (Ct) method by normalizing target mRNA Ct values to those of 18S RNA. The following PCR thermal cycling conditions were used: 3 min at 95 °C, and 40 cycles of 15 s at 95 °C and 58 s at 60 °C. All samples were run in triplicate.

### 2.5. Western Blot

Small pieces of fresh tumor tissue were homogenized by sonication in RIPA buffer (Pierce) supplemented with protease inhibitor cocktail (Sigma). An BCA protein assay kit (Pierce, Rockford, IL, USA) was used to determine the protein concentrations. Protein samples were diluted in 2× Laemmli buffer (Bio-Rad) and boiled for 10 min. Twenty micrograms of proteins from each sample were separated in 10% SDS-PAGE precast gels (Bio-Rad) and then transferred onto nitrocellulose membranes (Bio-Rad). Nonspecific binding sites on the membranes were blocked using 5% non-fat milk in Phosphate-Buffered Saline with Tween 20 (PBST). The membranes were then probed for primary antibodies against caspase 3 (CST #9665S), cleaved caspase 3 (CST #9661s), PARP1 (CST #95425), Ki67 (Abcam #ab15580), PCNA (CST #131105), or β-actin (Sigma, A2066), followed by the appropriate secondary antibody conjugated with horseradish peroxidase (HRP; Millipore). Protein detection was conducted using Pierce ECL Substrate (Pierce).

### 2.6. Hematoxylin and Eosin (H&E) Staining

Lungs were fixed and embedded in paraffin as described [24]. The lung tissues were cut into 5 μm sections and mounted on glass slides. Then, the slides were soaked twice for 10 min in xylene to deparaffinize the sections. The slides were passed through baths of decreasing ethanol concentrations: 90% ethanol, 80% ethanol, and 70% ethanol for 2 min, followed by double-distilled water for 1 min to hydrate the tissues. The tissues were stained in Mayer’s hematoxylin solution for 3 min and washed under running tap water for 10 min. The slides were then rinsed with 0.3% acid alcohol and counterstain in 1% eosin Y solution for 1 min. The excess eosin Y was removed by 2 changes in absolute alcohol, and checked under the microscope. The tissues were cleared by soaking the slides in xylene for 5 min. They were then sealed by adding a drop of the mounting medium and observed under the microscope.

### 2.7. Immunohistochemistry (IHC) Staining

The IHC staining was performed as per the manufacturer’s instructions using the Dako EnVision FLEX system as previously described [21]. IHC expression of PD-L1 (Thermo Fisher # PA5-20343), Ki-67 (Thermo Fisher # MA5-14520), or Caspase 3 (Thermo Fisher # 19677-1-AP) was performed on lung tissues which were soaked in 10% formalin for at least 24 h before being embedded in paraffin wax. The results were evaluated using Fiji ImageJ software (https://imagej.net/software/fiji/) and GraphPad Prism 8.

### 2.8. TUNEL Assay

The TUNEL assay was conducted according to the manufacturer’s instructions using the Click-iT™ TUNEL Colorimetric IHC Detection Kit (Invitrogen # C10625). In detail, the lung tissue sections were dewaxed and rehydrated. The slides were immersed in 4% paraformaldehyde for 15 min at 37 °C, which was followed by incubating them with permeabilization reagent (Proteinase K solution). The slides were again immersed in 4% paraformaldehyde and washed. After incubating the slides with TdT Reaction Buffer for 10 min at 37 °C, the TdT reaction was performed for 60 min at 37 °C. The TdT reaction was quenched by soaking the slides in 2× SSC and then washing them in PBS. Next, the slides were immersed in 3% H_2_O_2_ for 5 min at room temperature to quench endogenous peroxidase enzymes. The slides were then washed twice with 1× Click-iT™ TUNEL Colorimetric wash solution and incubated with the Click-iT™ TUNEL Colorimetric reaction cocktail for 30 min at 37 °C. After this, the slides were washed with PBS, then 1× Click-iT™ TUNEL Colorimetric wash solution, and deionized water. The slides were then incubated with 1× Streptavidin-Peroxidase Conjugate at room temperature for 30 min. After washing the slides with PBS and deionized water, the slides were developed with a 1× DAB reaction mixture. The reaction was stopped by immersing the slides in deionized water, counterstaining them with hematoxylin, mounting them, and scanning them with a microscope. Fiji ImageJ software and GraphPad Prism 8 were used to obtain data from the images for quantification and statistical analyses.

### 2.9. Statistical Analysis

The statistical analyses were performed using GraphPad Prism 8 and ImageJ. Quantitative values were showed as the mean ± SD. Differences between samples were analyzed by the ungrouped-samples *t* test. Pearson’s correlation coefficient was used to measure the expression level in IHC staining. The Kaplan–Meier survival curves were generated for survival comparison. *p* < 0.05 was considered statistically significant.

## 3. Results

### 3.1. VSV-S Suppresses Breast Cancer Lung Metastasis as a Neoadjuvant Therapy

**Figure 1:**
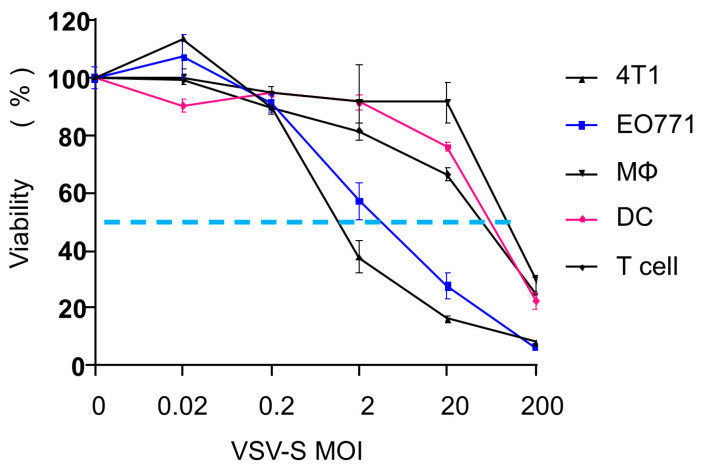
VSV-S infectivity in different cells. 4T1, EO771, macrophages, dendritic cells, and T cells were infected with VSV-S at different MOIs. Cell viability was measured 24 h after infection. The dotted line is drawn at 50% of viability.

4T1 and EO771 cells are mouse cancer cell lines used as common models for TNBC. We tested the cell selectivity of VSV-S in these two cell lines. Confluent cells were infected at various multiplicities of infection (MOI = 0, 0.002, 0.02, 0.2, 2, and 200). For comparison, macrophages, dendritic cells derived from mouse bone marrow, and CD3+ T cells from mouse spleen were also tested. As shown in Figure 1, both 4T1 and EO771 cells were effectively infected by VSV-S with a viability EC_50_ value of 2 MOI. In contrast, the EC_50_ values of VSV-S infection for macrophages, dendritic cells, and T cells were between 20 and 200 MOI, resulting in a selective ratio between 10 and 100. In our previous report, safety studies of VSV-S were carried out in mice with and without tumors [21]. Clinical chemistry, hematology, and coagulation analyses of blood samples confirmed that VSV-S has no detectable toxicity at a dose of 1.0 × 10^8^ PFU via intravenous injection. The overexpression of Smac was clearly confirmed in VSV-S-infected tumor cells, and infection of subcutaneous 4T1 tumors by VSV-S and its enhancement of capase-3 cleavage were established in a mouse model [20]. These studies suggest that VSV-S is a strong candidate for the treatment of TNBC models in mice.

**Figure 2:**
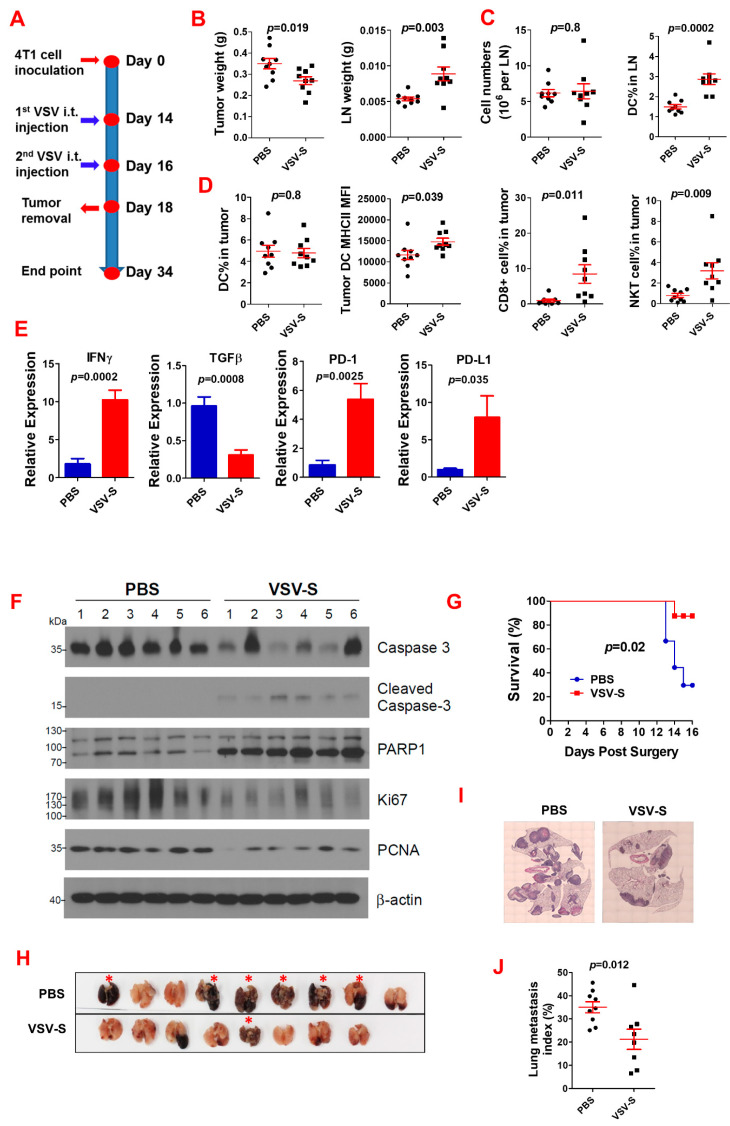
Neoadjuvant VSV-S treatment improves anti-tumor immunity and reduces post-surgery lung cancer metastasis. (**A**) Experimental design. N = 9 in the PBS control or VSV-S group. (**B**) The weights of tumors and the tumor-draining lymph nodes at the tumor removal. Red lines indicate the error ranges (the same in the following panels). (**C**) Total cell number and DC percentage in lymph nodes. (**D**) Immune cells in tumors. (**E**) Gene expression in tumors. (**F**) Western blot showing enhanced apoptosis and reduction in tumor cell proliferation by VSV-S. (**G**) Mouse survival after surgery. (**H**) The lungs of the mice at death before the endpoint or sacrifice. (**I**,**J**) Representative microscopic images of H&E-stained lung sections (**I**) and quantification (**J**) of lung metastasis. One mouse in the VSV-S group died at surgery. Red asterisks: mice died before the endpoint.

We first examined the impact of VSV-S as a neoadjuvant therapy on primary TNBC tumors and post-surgery cancer lung metastasis using a TNBC mouse model. The experimental design is shown in Figure 2A. Briefly, after 4T1 cell (2 × 10^5^) inoculation in each side of the 4th pair of mammary fat pads of 8-week-old female BLAB/c mice on Day 0, two doses of VSV-S (dose = 2 × 10^6^ PFU in 20 μL PBS for each tumor) were intratumorally (i.t.) injected on Days 14 and 16, the same volume of PBS was injected in control mice. The tumors were completely removed on Day 18, along with tumor-draining lymph nodes (LNs). The mice were then monitored for survival until Day 34. The lungs of mice that were euthanized at the endpoint or died before Day 34 were collected for analysis. VSV-S reduced the primary tumor weight and increased LN weight (Figure 2B). Flow cytometry analysis showed that VSV-S treatment increased dendritic cell (DC) numbers in LNs, while the total cell numbers in LNs were not different (Figure 2C). CD4+ T cells were not changed. Flow cytometry analysis also showed that VSV-S increased expression of MHC-II on DCs, and the number of CD8+ T and NKT cells in the tumors (Figure 2D). RT-qPCR revealed that IFNγ expression was increased while TGFβ expression was reduced in VSV-S-treated tumors; interestingly, VSV-S also significantly increased the expression of PD-1 and PD-L1 in the tumors (Figure 2E). The enhancement of apoptosis (increased cleaved caspase 3 and PARP1 levels) and reduction in tumor cell proliferation (decreased Ki67 and proliferating cell nuclear antigen (PCNA) levels) by VSV-S treatment were demonstrated by Western blot analysis of tumor tissues (Figure 2F). Survival analysis showed that VSV-S significantly improved mouse survival (Figure 2G). In the VSV-S-treated group, only one mouse among the eight mice died before the endpoint, with a long median survival of over 34 days, whereas in the PBS control group, six mice among the nine mice died before the endpoint, with a median survival of 14 days. Examination of the lungs shows that mice died before the endpoint due to severe cancer lung metastasis (Figure 2H–J). In a previous study, we showed that VSV-S treatment also reduced numbers of tumor-associated macrophages (TAMs), and myeloid-derived suppressor cells (MDSCs) [21].

### 3.2. Adaptation Increases the Selective Infectivity of VSV-S in 4T1 Cells

In our previous study, we demonstrated that adaptation by limited dilution could increase the selective VSV-S infectivity of mouse pancreatic cells by 20-fold [21]. Similarly, we carried out adaptation of VSV-S to 4T1 cells. The VSV-S stock grown in HeLa cells was serially diluted 10-fold in DMEM with 1% FBS and was used to infect the 90% confluent monolayers of 4T1 cells. The culture medium of the new virus stock was collected from the well in which cytopathic effect was observed at the highest dilution and was serially diluted for the next round of infection. After five rounds of adaptation, a stock of 4T1-adapted VSV-S was propagated and its titers were evaluated in comparison with a stock of VSV-S grown in HeLa cells (Table 1). As shown, the adaptation to 4T1 cells did not reduce VSV-S infectivity in HeLa cells. The titer by PFU/mL in the HeLa cells of the adapted VSV-S was 4-fold of the VSV-S grown in HeLa cells. However, the infectivity of the adapted VSV-S in 4T1 cells was enhanced by 600-fold, based on the TCID50/mL values. This confirms that adaptation by limited dilution could effectively increase the selective infectivity of VSV-S in 4T1 cells.

### 3.3. VSV-S Enhances the Efficacy of Immune Checkpoint Blockade

**Figure 3:**
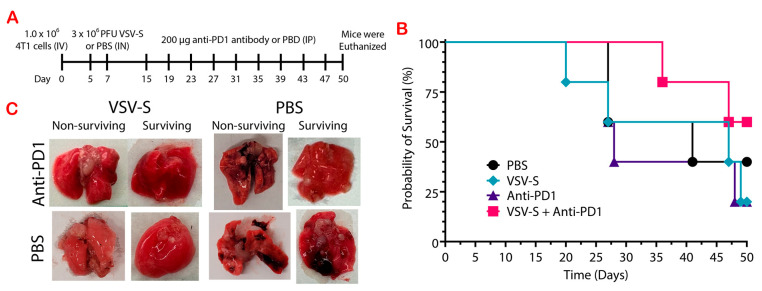
Treatment with adapted VSV-S in combination with anti-PD-1 decreased the size of tumors and the incidence of necrosis in a 4T1 metastatic model. (**A**) Experimental design. n = 5 in each of the 4 groups. (**B**) Mouse survival after 4T1 cell injections. (**C**) Representatives of the lung tissue removed from non-surviving and surviving mice from each group.

Previously we showed that VSV-S suppressed model tumors developed by subcutaneous injection of T47D and 4T1 cells in mice [20]. 4T1 cells are highly tumorigenic and spontaneously metastasize from the mammary gland to distant sites, such as lymph nodes, lungs, liver, and brain [25]. As shown in Figure 2E, VSV-S treatment enhanced the expression of PD-1 and PD-L1 in 4T1 tumors. We therefore tested the protective ability of VSV-S and VSV-S combined with anti-PD1 in a 4T1 metastatic model (Figure 3A). The 4T1-adapted VSV-S was used in this combination therapy since it increased the selective infection of 4T1 cells (Table 1). The mice were randomly assigned to four groups (n = 5): 1, VSV-S only; 2, VSV-S + anti-PD-1; 3, anti-PD-1 only; and 4, PBS. After injection of 1 × 10^6^ 4T1 cells via the tail vein, 3 × 10^6^ PFU VSV-S was administered intranasally in each mouse in groups 1 and 2 (n = 5) on Day 5, and 7, respectively. Equal volumes of PBS were administered intranasally in groups 3 and 4 as control. On Day 15 and every 4 days after that, the mice received an intraperitoneal (IP) injection of 200 μg of anti-PD1 antibody (BioXCell, RMP1-14, Lebanon, NH, USA) per mouse in groups 2 and 3, and equal volumes of PBS were injected (IP) in groups 1 and 4.

Survival was monitored and the experiment was terminated after 50 days, with more than half of group 2 (3/5) remaining alive. VSV-S combined with anti-PD1 improved the survival outcome, even though the difference in the overall survival was not statistically significant. This group has a median survival of greater than 50 days while the median survival for group 1 was 47 days (Figure 3B). In contrast, the median survival was only 28 days for group 3. Treatment with anti-PD-1 only in group 3 did not increase the survival rate in comparison with control group 4. Treatment with the combination therapy of VSV-S/anti-PD-1 greatly increased the probability of survival in this tumor model. Most notable is the improved quality of the lung tissue in groups 1 and 2. Group 2 lungs did not contain noticeable surface tumors and only one lung had small regions of necrosis (Figure 3C). Group 1 lungs had visible tumors on the surface of the lungs, and necrotic tissue. However, the incidence of necrosis was much less in group 3 than in group 1, showing that VSV-S greatly reduces the incidence of necrosis in lung tissue, and decreases the size of tumors when combined with anti-PD1. All of the mice in group 4 had multiple, visible tumors on the surface of the lung and large areas of necrotic tissue.

In addition, a number of the mice developed tumors (untreated) on their backs correlating to the location of the lumbar lymph nodes. On day 27, two mice in the PBS group, two in the anti-PD-1 group, and one mouse in the VSV-S group were euthanized when these lumbar tumors exceeded 2 cm. At the end of the experiment (day 50) a second mouse in the VSV-S group and only one in the VSV-S + anti-PD-1 group had developed a lumbar tumor that was less than 1 cm in length. Hence, VSV-S in combination with anti-PD-1 decreases the size and incidence of developing secondary lumbar tumors.

**Figure 4:**
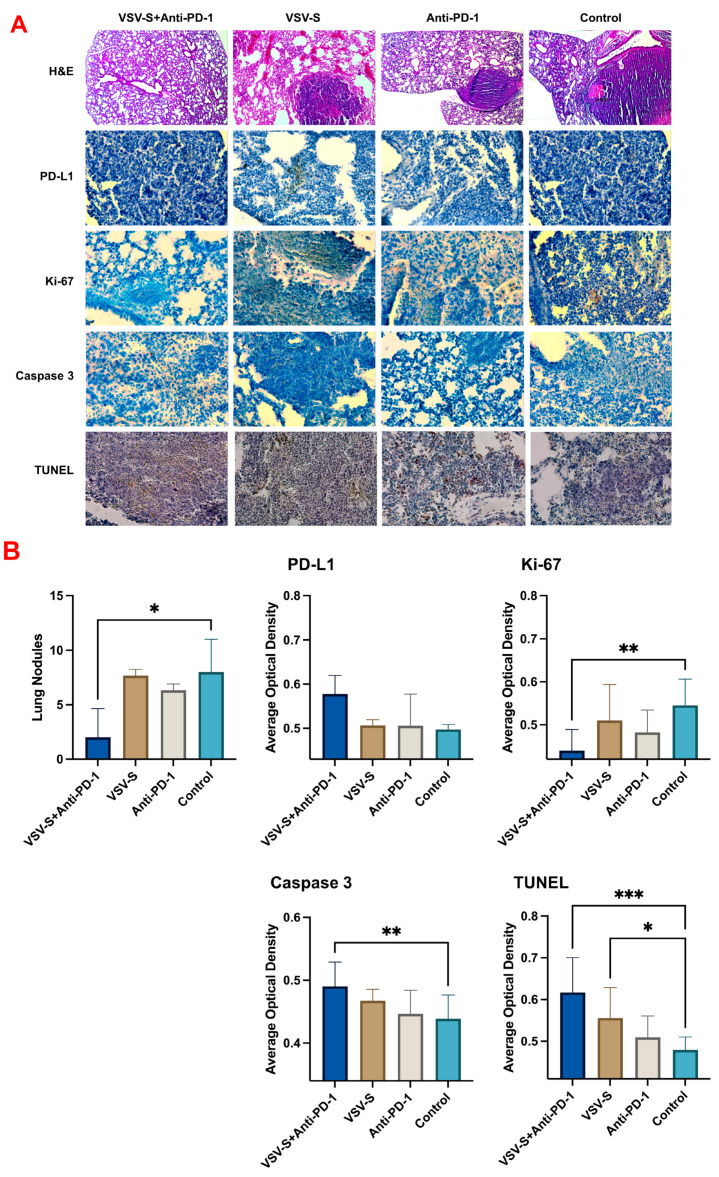
Combination therapy reduced tumor progression and promoted apoptosis. (**A**) Representative images of hematoxylin and eosin (H&E) staining, immunohistochemistry (IHC) staining for PD-L1, Ki-67, overall Caspase 3 and the colorimetric TUNEL assay (n = 5/group), (**B**) bar graphs of the lung nodule count and ImageJ analysis of the staining results. Quantitative values were showed as the mean ± SD. * *p* < 0.05, ** *p* < 0.01; *** *p* < 0.001.

The underlining mechanism for the positive outcomes of VSV-S + anti-PD-1 combination therapy was assessed by analyses of the tumor samples (Figure 4). The lung nodules in each group were counted. Control group 4 showed the largest solid tumor legions inside the lung. Group 1, treated with VSV-S only, had fewer tumor modules compared to the control group. The tumor legions showed a clear boundary with the surrounding tissues. There was degeneration and necrosis within the tumors as well. The lung was infiltrated with immune cells, predominantly consisting of neutrophils and lymphocytes according to their morphology. Group 3, treated with anti-PD-1 only, also had fewer tumor modules. There was no significant immune cell infiltration. Group 2, treated with both VSV-S and anti-PD-1, had the fewest tumor modules compared to the other groups. The morphology and structure of the bronchi, alveoli, and alveolar septa were normal. The bronchial mucosal epithelium was intact, with clear alveolar ducts and alveolar structures. The alveoli were neither dilated nor collapsed, and the alveolar septa was not thickened. We found that both the VSV-S and anti-PD-1 treatments restricted the tumors, but the combination therapy resulted in the best outcomes.

To better evaluate the metastasis of the tumor cells in the lung, immunohistochemistry staining (IHC) and terminal deoxynucleotidyl transferase dUTP nick-end labeling (TUNEL) assays were performed (Figure 4). PD-1 is mainly expressed on T cells, whereas PD-L1 is expressed on cancer cells and antigen presenting cells [26]. Combination therapy increased the expression level of PD-L1 compared to the monotherapies and the control group. The proportion of the proliferation marker Ki-67 was significantly reduced following the combination therapy compared to the control group. The combination therapy group has higher caspase-3 levels than the monotherapy group, or the control group, indicating the induction of apoptosis [27,28]. Furthermore, there was a significant increase in positive TUNEL staining in both the VSV-S-treated group and the combination therapy group. All the data above prove that the combination therapy induced apoptosis in the tumor and decreased the metastasis in the lung.

## 4. Discussion

Treatment for breast cancer has been dramatically improved in recent years, with reassuring prognosis of early-stage disease achieved by targeted and adjuvant therapies. For further progress, immunotherapy is a promising new avenue for breast cancer treatment, especially for late-stage or metastatic disease [29]. In the case of TNBC, the efficacy of immunotherapy is heavily dependent on the tumor microenvironment (TME) [30]. In our previous studies, we have demonstrated that treatment with our Smac-armed VSV-S changed the TME [21]. The infiltration of CD8+ T cells and neutrophils was greatly increased, while the number of M2 macrophages and MDSCs were significantly reduced, indicating the reversion of an immunosuppressive (cold) TME to an immunosensitive (hot) TME. In this study, we applied VSV-S to TNBC treatment using two animal models.

In the neoadjuvant treatment model, primary tumors generated by implanting 4T1 cells in the mammary fat pads, were treated with the intratumoral injection of VSV-S. The purpose of VSV-S treatment is mainly aimed at reverting the TME, not directly eliminating the tumor mass. Since VSV-S could cause local inflammation and release tumor neoantigens by oncolysis, this treatment of primary tumors would induce systematic immune responses to 4T1 tumors. As the results show, we observed a significant enhancement of post-surgery survival due to an evident reduction in lung metastasis after VSV-S treatment and subsequent primary tumor surgical removal. Similar results were also observed in treatment with other OVs [31]. In addition, changes in cell populations in the tumors, including LN dendritic cells, CD8+ T, and NKT cells, also pointed to a positive antitumor response of the systematic immunity, further supported by an increase in IFNγ expression and an increase in IFNγ expression. Other results are also consistent with our conclusions [32,33]. More interestingly, the level of PD-1 and PD-L1 expression in tumors was increased by VSV-S treatment, suggesting that if VSV-S is combined with anti-PD-1 treatment, VSV-S may enhance the efficacy of the latter.

In our follow-up study in the second animal model of lung metastatic 4T1 tumors, we applied VSV-S/anti-PD-1 combination therapy. Before the application of the combination treatment, we enhanced selective infection of 4T1 cells by VSV-S through a unique adaptation approach of limited dilution. By limited dilution adaptation, the evolved VSV-S has a virulent property of rapid expansion in 4T1 cells, not just a higher yield as those selected by the conventional adaptation procedures. This 4T1-adapted VSV-S is likely to increase the neoantigen release and TME changes after tumor treatment due to its increased selective infection. In the metastatic model generated by tail vein injection of 4T1 cells, lung tumors were treated by intranasal VSV-S. This delivery route allowed us to achieve a relatively higher concentration of VSV-S in the lung and, in addition, the 4T1 adaptation also makes it possible to use a lower dosage of VSV-S to solicit the positive responses. The treatment of anti-PD-1 via IP injection was given 8 days after intranasal VSV-S treatment, allowing ample time for full activation of systematic immune responses before the administration of the anti-PD-1 antibody. As the results showed, combination therapy of VSV-S and anti-PD-1 antibody has significant improvements over VSV-S or anti-PD-1 antibody alone, even though the sole treatment also showed efficacy to some extent. A long median survival and improved lung pathology after combination therapy clearly demonstrated the enhanced efficacy of immunotherapy. Detailed analyses of the treated lungs (Figure 4) confirmed the conclusion.

## 5. Conclusions

The key advances in our study include increases in selective infection of tumor cells by VSV-S through limited dilution adaptation. The adapted VSV-S allows us to adequately dose VSV-S in the lungs via intranasal delivery. The effective pulmonary delivery of an oncolytic virus solves a persistent problem in the field because of the difficulty to effectively deliver the oncolytic virus to the tumor site. If an oncolytic virus is delivered systematically, very high doses are required and a large portion of the administered oncolytic virus is quickly cleared from circulation before adequate distribution in the tumor site. Pulmonary delivery directly administers the oncolytic virus in the lung to achieve high local concentrations, and the tumor adapted oncolytic virus effectively infects the tumor cells even at a relatively low dose. The effectiveness of this delivery route is verified by the enhancement of the efficacy of immunotherapy.

## 6. Patents

WO2018213412A1—Recombinant oncolytic virus has been filed for the armed vesicular stomatitis virus (VSV-S).

## Figures and Tables

**Table 1:** Infectivity of 4T1 adapted VSV-S.

VSV-S Stock	TCID50/mL (4T1 Cells)	PFU/mL (HeLa Cells)
adapted to 4T1 cells	7.92 ± 1.01 × 10^7^	3.13 ± 0.2 × 10^7^
grown in HeLa cells	1.32 ± 0.32 × 10^5^	8 ± 0.5 × 10^6^

## Data Availability

The data presented in this study are available on request from the corresponding author.
